# Comparison of the prognostic validity of three simplified consciousness assessment scales with the Glasgow Coma Scale

**DOI:** 10.1007/s00068-023-02286-w

**Published:** 2023-06-09

**Authors:** Dimitrios M. Anestis, Konstantinos Marinos, Parmenion P. Tsitsopoulos

**Affiliations:** Department of Neurosurgery, Hippokration General Hospital, Aristotle University School of Medicine, 49 Konstantinoupoleos str., 54642 Thessaloníki, Greece

**Keywords:** AVPU scale, Glasgow coma scale, Modified Glasgow coma scale motor response, Simplified motor scale, Prognostic value

## Abstract

**Background:**

Various tools simpler than the Glasgow Coma Scale (GCS) have been proposed for the assessment of consciousness. In this study, the validity of three coma scales [Simplified Motor Scale, Modified GCS Motor Response, and AVPU (alert, verbal, painful, unresponsive)] is evaluated for the recognition of coma and the prediction of short- and long-term mortality and poor outcome. The predictive validity of these scales is also compared to the GCS.

**Methods:**

Patients treated in the Department of Neurosurgery and the Intensive Care Unit in need of consciousness monitoring were assessed by four raters (two consultants, a resident and a nurse) using the GCS. The corresponding values of the simplified scales were estimated. Outcome was recorded at discharge and at 6 months. Areas Under the Receiver Operating Characteristic Curve (AUCs) were calculated for the prediction of mortality and poor outcome, and the identification of coma.

**Results:**

Eighty-six patients were included. The simplified scales showed good overall validity (AUCs > 0.720 for all outcomes of interest), but lower than the GCS. For the identification of coma and the prediction of long-term poor outcome, the difference was significant (*p* < 0.050) for all the ratings of the most experienced rater. The validity of these scales was comparable to the GCS only in predicting in-hospital mortality, but without this being consistent for all raters.

**Conclusion:**

The simplified scales showed inferior validity than the GCS. Their potential role in clinical practice needs further investigation. Thus, the replacement of the GCS as the main scale for consciousness assessment cannot be currently supported.

## Introduction

The Glasgow Coma Scale (GCS) [1, 2] has been widely accepted as the gold standard for assessing the level of consciousness and the evaluation of the depth of coma [3, 4]. Despite its success, the scale has received criticism for its potential drawbacks [3, 5, 6]. Thus, various alternative clinical tools have been proposed [3, 6, 7]. Among them, it has been suggested that several scales less complex than the GCS can achieve a faster, but equally reliable patient assessment with similar predictive accuracy, especially during their application in the pre-hospital and emergency department setting [8–13]. Thus, the potential replacement of the GCS has been proposed in the literature [14].

In the current study, three simplified consciousness assessment systems were studied, namely the Simplified Motor Scale (SMS) [8], the Modified GCS Motor Response (MGMR) [12], and the AVPU scale (alert, verbal, painful, unresponsive) [10]. In particular, the study aimed: (1) to evaluate the validity of these scales in recognizing the comatose condition, (2) to evaluate their prognostic validity in predicting short- and long-term poor outcomes, and (3) to compare the prognostic validity of the simpler scales to the GCS.

## Methods

### Design & setting

A prospective observational study was carried out following the STROBE cohort reporting guidelines [15]. The study was conducted at Hippokration General Hospital, Thessaloniki, Greece, between October 1st, 2018 and December 31st, 2020. Cases with neurosurgical pathologies managed in the Department of Neurosurgery (a 24-bed unit, which also hospitalizes patients who require intensive care and closer monitoring) and the Intensive Care Unit (ICU) were included.

Participants’ clinical and radiological data during hospitalization were collected. Level of consciousness was recorded on admission and in case of clinical (neurological) deterioration. Outcome assessment was obtained at discharge and at 6 months.

All authors agreed with the study protocol which was approved by the hospital’s Ethics Committee (Ref. Nr. 985-2017). The National Data Protection Authority was also informed (Ref. Nr. 850-2018). Legal consent was obtained from all patients or by proxy when deemed necessary. The ethical standards of the 1964 Helsinki Declaration and its subsequent modifications were followed.

### Variables & data collection

The level of consciousness was assessed with the application of the GCS on admission (within 12 h from presentation) by the following four raters: a senior consultant neurosurgeon (author PPT), a junior consultant neurosurgeon (author DMA), a neurosurgery resident (6 in total, covering all years of training) and a registered nurse (8 in total, all with at least 10 years of experience). All ratings were blinded and performed independently within 1 h at maximum. In case of clinical deterioration resulting from acute brain damage, a full rating session was reiterated, and only the updated assessment was used for the estimations.

Outcome was assessed with the Modified Rankin Scale (mRS) [16, 17] and the Glasgow Outcome Scale-Extended (GOSE) [18, 19]. For the estimation of the 6-month outcome, patients were evaluated through phone calls [20, 21]. To avoid bias, outcome assessments were blinded to any consciousness assessments. For the identification of coma, patients were categorized as comatose before consciousness assessments, based on the standard definition of coma given by Frowein (eyes continuously closed, only reflex or defense movements, with or without stimuli) [22]. Since the validity of the simpler scales in diagnosing coma was compared to that of the GCS, using the total GCS score as a criterion for coma would have been subject to bias.

Three simplified consciousness assessment models that have been previously studied in the literature were selected (Table 1): (1) the SMS, which uses the GCS motor component to categorize patients into three groups (obeying, localizing and less) [8], (2) the MGMR, which is similar to the SMS but with different categorization (obeying, not obeying and not responding) [12], and (3) the AVPU scale, which in the “alert” category includes patients with eyes spontaneously open, orientated speech and obeying commands, in the “verbal” and “painful” categories those with any verbal, motor or eye response to a verbal or painful stimulus respectively, and in the “unresponsive” category those that do not respond; the algorithm provided by Kelly et al. was used for calculations [10]. These models were not directly used for patient assessments, but their values were calculated based on the GCS recordings, a method that has been previously used in similar studies [8, 12, 13, 23].

**Table 1:** The simplified systems for the assessment of consciousness that were studied, and their definition based on the GCS [8, 10, 12]

Scale	Value	Definition based on GCS
SMS	2	GCS motor component = 6
	1	GCS motor component = 5
	0	GCS motor component = 1–4
MGMR	2	GCS motor component = 6
	1	GCS motor component = 2–5
	0	GCS motor component = 1
AVPU	A	GCS total score = 15
	V	Not A, P or U
	P	GCS total score = 4–14
		And GCS eye component = 1–2
		And GCS verbal component = 1
		And GCS motor component = 1–5
	U	GCS total score = 3

*AVPU* alert/verbal/painful/unresponsive scale, *GCS* Glasgow coma scale, *MGMR* modified GCS motor response, *SMS* simplified motor scale

Since the AVPU scale does not include a particular scoring system nor any has been previously reported [9–11, 13, 24], the following values were used for calculations, in concurrence with the other included models: 3 for alert, 2 for verbal, 1 for painful and 0 for unresponsive. The ACDU scale (alert, confused, drowsy, unresponsive) was not included in this study, because its components are not clearly defined [9, 11].

Data were collected directly during hospitalization, they were totally anonymized and digitally documented in a Microsoft Excel© 2019 (Microsoft Corporation, Redmond, Washington, USA) worksheet. Assessment values according to the simplified systems were automatically calculated (utilizing built-in software functions) based on the recorded GCS values. The procedure fully complied with the current legislation.

### Eligibility criteria

Inclusion criteria were: (1) age ≥ 18 years old, (2) need for neurosurgical care, constant clinical assessments by a neurosurgeon, and possible intervention, (3) hospitalization in the Neurosurgery Department and/or the ICU, and (4) impaired level of consciousness; to avoid bias, patients with initially normal responsiveness but in need for consciousness monitoring due to risk of neurologic deterioration were also included.

Patients having one of the following were excluded: (1) failure to obtain legal consent, (2) unavailability of all examiners to obtain a reliable and blinded clinical assessment within 1 h, (3) inability to obtain a complete patient assessment within 12 h from presentation, (4) failure to record the worst neurological picture, 5) failure to obtain outcome at discharge and/or at 6 months, (6) conditions and agents that would influence the reliability of the assessments, (e.g., mental diseases, dementia, sedatives, neuromuscular junction blockers, and addictive substances), and (7) missing data. ICU patients were only assessed after the administration of any of the aforementioned agents was stopped long enough to eliminate its effect according to the ICU protocols, so that a reliable clinical assessment could be obtained [25].

To avoid bias, in the event of clinical deterioration the worst recorded values were used, only if this worsening was directly linked to the main pathology.

### Statistical analysis

Descriptive statistics are presented as means ± standard deviation or medians. Normality of data was checked with the Kolmogorov–Smirnov test. *p* values < 0.050 were considered statistically significant.

The outcomes of interest were mortality at discharge (in-hospital mortality) and at 6 months (long-term mortality), poor outcome at discharge (short-term outcome) and at 6 months (long-term outcome). Poor outcome was defined as mRS values of 3 to 6 and GOSE values of 1–4 [26–28].

Areas Under the Receiver Operating Characteristic Curve (AUCs) for all simplified assessment systems and in comparison with the GCS were calculated for each rater, as previously reported [25]. This way of assessing coma scales is widely accepted and has been used before [8, 12, 23, 29, 30]. The formula proposed by de Long [31] was followed for AUC comparisons [28, 32–35], with Bonferroni correction for multiple comparisons [36, 37]. AUCs for all outcomes of interest were also calculated for a sub-group analysis, including only patients with head trauma.

A power analysis was carried out to define the minimum number of participants that would reach an adequate statistical strength. AUC values of approximately 0.900 were expected, and the level of difference between them was set at 5%. It was found that 18 subjects for AUC calculations and 84 subjects for comparisons would reach a power of 80% and a 5% level of significance, which were considered appropriate for the purposes of the current study [25, 38].

The software package MedCalc© version 20 (MedCalc Software Ltd, Ostend, Belgium) was used for the statistical analysis.

## Results

Among the 489 eligible patients, 86 were finally enrolled. None was excluded due to missing data. Most were men (61.6%), with a median age of 73.5 years and a hospitalization of 15 days. The majority presented with acute head trauma (41.9%). Forty (46.5%) died during hospitalization while 51 (59.3%) were dead at 6 months (Table 2). Patients’ assessments with their corresponding GCS values are shown in Table 3. Tables 4 and 5 present the AUC values for the included scales, for each outcome of interest and rater, and in comparison with the GCS.

**Table 2:** Demographic data of the participants

Characteristic	Value	%
Number of cases	86	
Male/Female	53/33	61.6/38.4
Age (y)	73.5 (18–97)	
Length of stay (d)	15 (1–137)	
Diagnosis		
Acute head trauma	36	41.9
Ischemic/Hemorrhagic stroke	19	22.1
Chronic/Subacute SDH	15	17.4
Aneurysmal SAH	6	7.0
Neoplasia	5	5.8
Hydrocephalus	4	4.7
CNS Infection	1	1.2
Hospitalization in the ICU	33	38.4
Length of stay (d)	16 (1–57)	
Mechanical Ventilation	32	37.2
Stay on ventilator (d)	14 (1–50)	
Mortality		
At discharge	40	46.5
At 6 months	51	59.3

*CNS* central nervous system, *d* days, *ICU* intensive care unit, *SAH* subarachnoid hemorrhage, *SDH* subdural hematoma, *y* years

**Table 3:** Assessment results with the models included in the study and their corresponding GCS values

Scale	Value	*N*	%	Corresponding GCS Values	
				Range	Median
SMS	2	41	47.7	9–15	12
	1	9	10.5	7–12	11
	0	36	41.9	3–9	6
MGMR	2	41	47.7	9–15	12
	1	38	44.2	4–12	7
	0	7	8.1	3–6	3
AVPU	A	4	4.7	15	15
	V	42	48.8	5–14	12
	P	35	40.7	4–11	7
	U	5	5.8	3	3

*AVPU* alert/verbal/painful/unresponsive scale, *GCS* Glasgow coma scale, *MGMR* modified GCS motor response, *SMS* simplified motor scale

**Table 4:** AUC values with 95% CI for identifying coma and short-term mortality and poor outcome, for each scale and rater. The results from the comparison with the GCS are also shown

Rater	Senior Consultant			Junior Consultant			Resident			Nurse		
	AUC	95% CI	*p*	AUC	95% CI	*p*	AUC	95% CI	*p*	AUC	95% CI	*p*
Coma identification												
GCS	0.968^a^	0.906–0.994	–	0.976^a^	0.918–0.997	–	0.954^a^	0.886–0.988	–	0.968^a^	0.906–0.994	–
SMS	0.903^a^	0.820–0.957	0.005*	0.892^b^	0.807–0.949	< 0.001*	0.887^b^	0.800–0.945	0.010*	0.890^b^	0.805–0.948	0.002*
MGMR	0.876^b^	0.788–0.937	< 0.001*	0.853^b^	0.761–0.920	< 0.001*	0.862^b^	0.771–0.927	0.001*	0.860^b^	0.768–0.925	< 0.001*
AVPU	0.891^b^	0.806–0.948	0.015*	0.908^a^	0.826–0.959	0.003*	0.881^b^	0.793–0.941	0.002*	0.901^a^	0.818–0.955	0.004*
In-hospital mortality												
GCS	0.923^a^	0.845–0.969	–	0.906^a^	0.823–0.958	–	0.886^b^	0.800–0.945	–	0.924^a^	0.847–0.970	–
SMS	0.852^b^	0.759–0.919	0.005*	0.873^b^	0.784–0.935	0.397	0.859^b^	0.767–0.924	0.720	0.882^b^	0.794–0.941	0.089
MGMR	0.826^b^	0.730–0.900	0.002*	0.865^b^	0.774–0.929	0.422	0.846^b^	0.752–0.915	0.400	0.852^b^	0.759–0.920	0.015*
AVPU	0.833^b^	0.737–0.904	0.002*	0.876^b^	0.787–0.937	0.539	0.834^b^	0.739–0.906	0.125	0.893^b^	0.808–0.950	0.336
mRS 3–6 at discharge												
GCS	0.935^a^	0.861–0.977	–	0.905^a^	0.823–0.958	–	0.931^a^	0.855–0.974	–	0.928^a^	0.852–0.973	–
SMS	0.770^c^	0.666–0.854	< 0.001*	0.840^b^	0.746–0.910	0.056	0.833^b^	0.737–0.904	< 0.001*	0.834^b^	0.738–0.905	0.001*
MGMR	0.734^c^	0.628–0.824	< 0.001*	0.829^b^	0.733–0.902	0.047*	0.821^b^	0.724–0.895	< 0.001*	0.822^b^	0.725–0.896	< 0.001*
AVPU	0.808^b^	0.709–0.885	< 0.001*	0.842^b^	0.748–0.912	0.025*	0.860^b^	0.768–0.925	0.008*	0.907^a^	0.825–0.959	0.505
GOSE 1–4 at discharge												
GCS	0.911^a^	0.830–0.962	–	0.883^b^	0.796–0.943	–	0.928^a^	0.851–0.972	–	0.916^a^	0.836–0.965	–
SMS	0.765^c^	0.661–0.850	< 0.001*	0.834^b^	0.738–0.906	0.222	0.845^b^	0.751–0.914	0.002*	0.846^b^	0.752–0.915	0.021*
MGMR	0.721^c^	0.614–0.813	< 0.001*	0.814^b^	0.715–0.889	0.097	0.834^b^	0.738–0.906	< 0.001*	0.835^b^	0.739–0.906	0.014*
AVPU	0.784^c^	0.682–0.866	< 0.001*	0.818^b^	0.720–0.893	0.047*	0.834^b^	0.738–0.906	< 0.001*	0.902^a^	0.818–0.955	> 0.999

*AUC* area under the receiver operating characteristic curve, *AVPU* alert/verbal/painful/unresponsive scale, *CI* confidence interval, *GCS* Glasgow coma scale, *GOSE* Glasgow outcome scale-extended, *mRS* modified Rankin scale, *MGMR* modified GCS motor response, *p* *p* values for the comparison with the GCS, *SMS* simplified motor scale

*Denotes statistically significant difference (*p* < 0.050)

^a^Denotes excellent predictive validity

^b^Denotes very good predictive validity

^c^Denotes good predictive validity

**Table 5:** AUC values with 95% CI for long-term mortality and poor outcome, for each scale and rater. The results from the comparison with the GCS are also shown

Rater	Senior Consultant			Junior Consultant			Resident			Nurse		
	AUC	95% CI	*p*	AUC	95% CI	*p*	AUC	95% CI	*p*	AUC	95% CI	*p*
6-Month mortality												
GCS	0.923^a^	0.845–0.969	–	0.918^a^	0.838–0.966	–	0.910^a^	0.828–0.961	–	0.939^a^	0.865–0.979	–
SMS	0.852^b^	0.759–0.919	0.003*	0.857^b^	0.765–0.923	0.040*	0.846^b^	0.752–0.915	0.062	0.891^b^	0.806–0.948	0.097
MGMR	0.826^b^	0.730–0.900	< 0.001*	0.853^b^	0.760–0.920	0.051	0.839^b^	0.745–0.910	0.031*	0.864^b^	0.773–0.929	0.017*
AVPU	0.833^b^	0.737–0.904	< 0.001*	0.854^b^	0.762–0.921	0.017*	0.853^b^	0.760–0.920	0.047*	0.899^b^	0.816–0.954	0.147
6-Month mRS 3–6												
GCS	0.947^a^	0.876–0.984	–	0.959^a^	0.892–0.990	–	0.960^a^	0.895–0.991	–	0.962^a^	0.896–0.991	–
SMS	0.849^b^	0.756–0.917	0.002*	0.885^b^	0.799–0.944	0.009*	0.891^b^	0.805–0.948	0.021*	0.893^b^	0.807–0.949	0.005*
MGMR	0.823^b^	0.726–0.897	< 0.001*	0.884^b^	0.797–0.943	0.010*	0.874^b^	0.785–0.936	0.005*	0.875^b^	0.786–0.937	0.001*
AVPU	0.848^b^	0.755–0.916	< 0.001*	0.881^b^	0.793–0.941	0.001*	0.881^b^	0.793–0.941	0.002*	0.913^a^	0.833–0.963	0.022*
6-Month GOSE 1–4												
GCS	0.955^a^	0.888–0.988	–	0.970^a^	0.908–0.995	–	0.971^a^	0.910–0.995	–	0.969^a^	0.907–0.994	–
SMS	0.842^b^	0.747–0.912	< 0.001*	0.907^a^	0.825–0.959	0.025*	0.863^b^	0.796–0.942	0.002*	0.899^b^	0.815–0.954	0.005*
MGMR	0.815^b^	0.717–0.891	< 0.001*	0.899^b^	0.815–0.954	0.020*	0.864^b^	0.775–0.930	< 0.001*	0.890^b^	0.805–0.948	0.003*
AVPU	0.843^b^	0.749–0.913	< 0.001*	0.898^b^	0.814–0.953	0.004*	0.865^b^	0.787–0.937	< 0.001*	0.931^a^	0.855–0.974	0.047*

*AUC* area under the receiver operating characteristic curve, *AVPU* alert/verbal/painful/unresponsive scale, *CI* confidence interval, *GCS* Glasgow coma scale, *GOSE* Glasgow outcome scale-extended, *mRS* modified Rankin scale, *MGMR* modified GCS motor response, *p* *p* values for the comparison with the GCS, *SMS* simplified motor scale

*Denotes statistically significant difference (*p* < 0.050)

^a^Denotes excellent predictive validity

^b^Denotes very good predictive validity

^c^Denotes good predictive validity

### Identification of coma

**Fig. 1:**
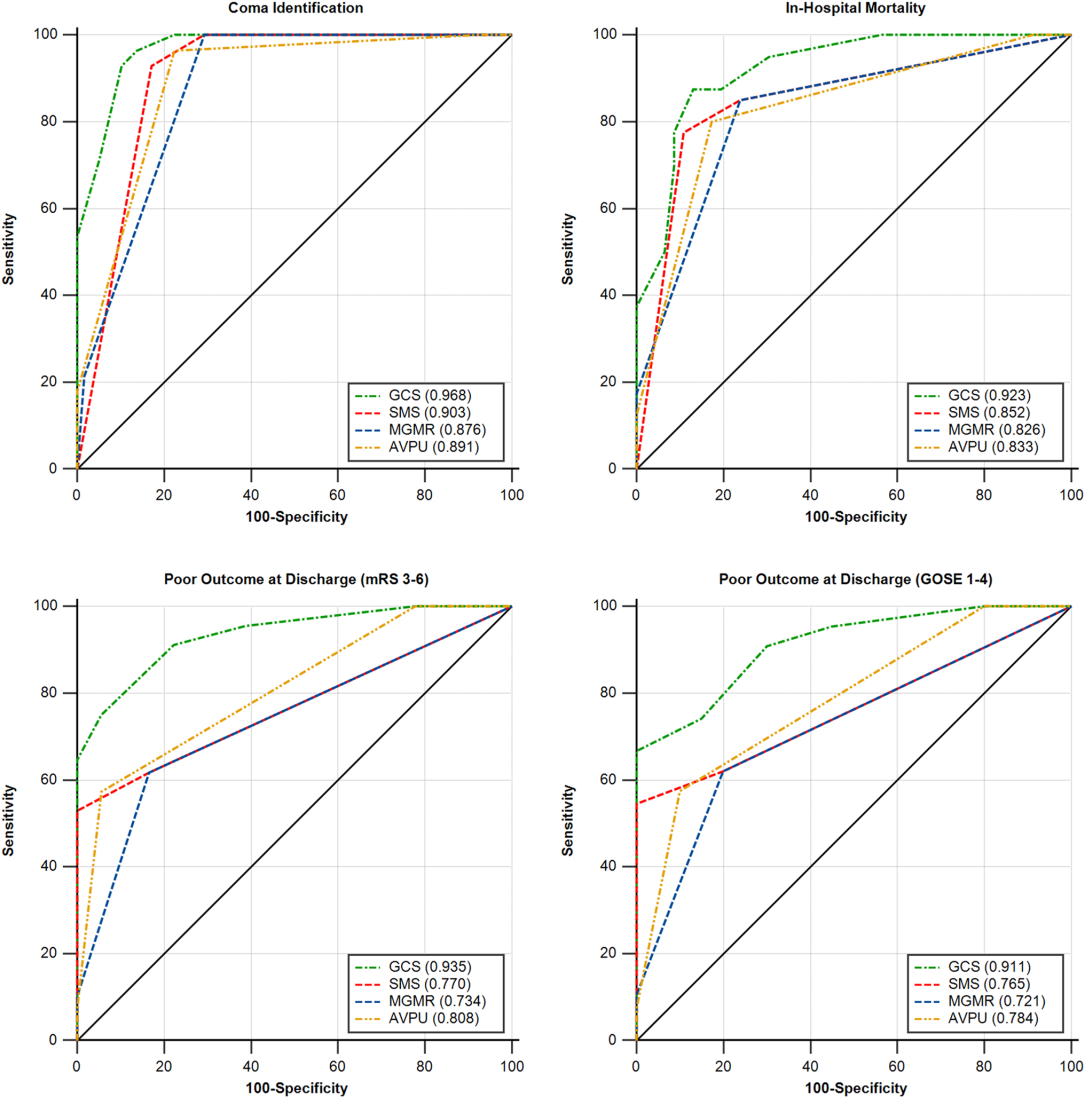
AUC values of the GCS and the three simplified scales (AVPU, MGMR & SMS) that were included in the study for identifying coma and predicting short-term mortality and poor outcome, according to the assessments of the senior consultant. The results were significantly higher for the GCS compared to the simplified scales (*p* < 0.050). *AUC* area under the receiver operating characteristic curve, *AVPU* alert/verbal/painful/unresponsive scale, *GCS* Glasgow coma scale, *GOSE* Glasgow outcome scale-extended, *mRS* modified Rankin scale, *MGMR* modified GCS motor response, SMS, simplified motor scale

Regarding the ability of identifying coma, the validity of the simplified scales was at least very good (AUCs > 0.850) in all cases, but significantly lower than the GCS (*p* < 0.050 in all comparisons, Table 4 and Fig. 1).

### Short-term outcomes

All simplified scales showed very good validity in predicting in-hospital mortality (AUCs = 0.825–0.893, Table 4 and Fig. 1), however, lower than the GCS. Notably, the difference was significant for the ratings of the senior consultant, but there was no significant difference for the other raters, with the exception of the MGMR according to the nurses’ ratings (Table 4).

As per the poor outcome at discharge, all simplified scales showed at least good prognostic validity (AUCs > 0.720, Table 4 and Fig. 1) in all occasions. Once again, the GCS results demonstrated higher prognostic validity for every rater and in comparison with any of the studied scales, but the differences were only sporadically non-significant. Specifically, the GCS presented with significantly higher values compared to any other scale for the assessments of the senior consultant and the residents. The difference was not significant for the AVPU according to the nurses, the SMS for the junior consultant for both poor outcome definitions, and the MGMR according to the junior consultant only for the GOSE 1–4 definition of poor outcome (Table 4).

### Long-term outcomes

**Fig. 2:**
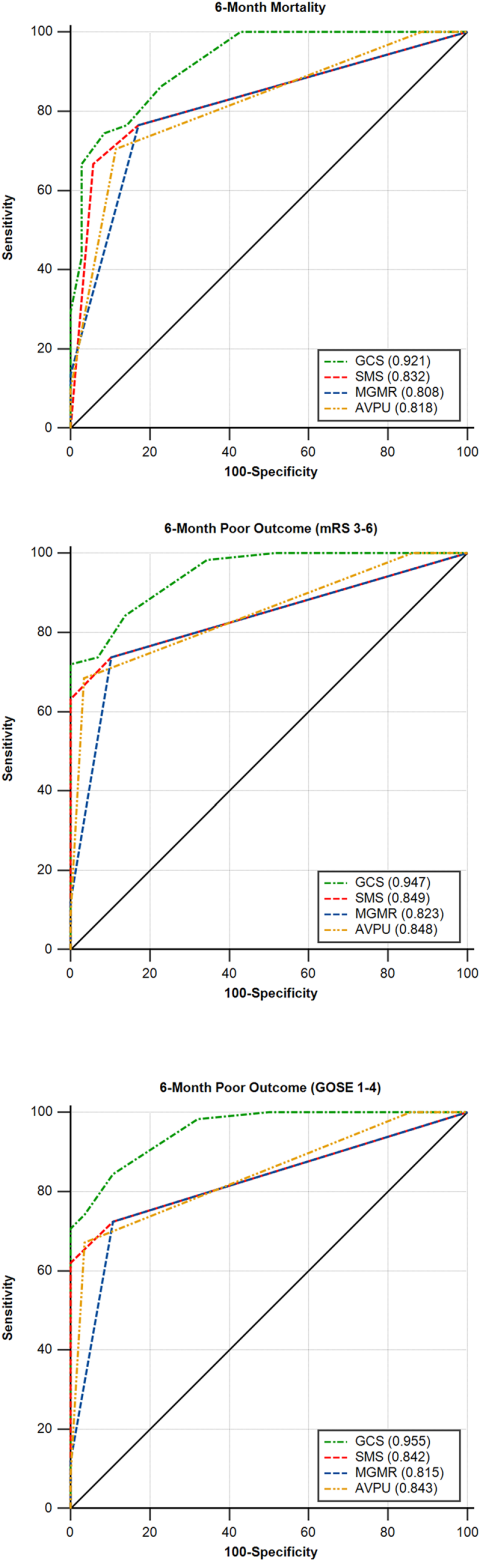
AUC values of the GCS and the three simplified scales (AVPU, MGMR & SMS) that were included in the study for predicting long-term mortality and poor outcome, according to the assessments of the senior consultant. The results were significantly higher for the GCS compared to the simplified scales (*p* < 0.050). *AUC* area under the receiver operating characteristic curve, *AVPU* alert/verbal/painful/unresponsive scale, *GCS* Glasgow coma scale, *GOSE* Glasgow outcome scale-extended, *mRS* modified Rankin scale, *MGMR* modified GCS motor response, SMS, simplified motor scale

The simplified scales showed at least very good validity for the prediction of mortality and poor outcome at 6 months (AUCs > 0.810 in all cases, Table 5 and Fig. 2). The corresponding GCS values were higher without any exception. The differences were significant in all cases (*p* < 0.050), except for the prediction of long-term mortality, for the SMS according to the residents and the nurses, the MGMR according to the junior consultant, and the AVPU according to the nurses (Table 5).

### Sub-group analysis

Fifty-one patients presented with head trauma (Table 2), and were included in the sub-group analysis. The results are presented in the Supplementary Table.

For the identification of coma, the GCS showed excellent validity with very high AUC values (> 0.960), while the other scales showed at least very good validity (AUCs > 0.885) in all cases and excellent (AUCs > 0.900) in the majority of them (Supplementary Table).

For in-hospital mortality, the short scales showed at least very good validity (AUCs = 0.838–0.905), whereas in predicting short-term outcomes the results were lower, with their validity considered as merely good (AUCs < 0.800) in most cases (AUCs = 0.703–0.878 for mRS 3–6, and 0.667–0.874 for GOSE 1–4, Supplementary Table). The GCS values were higher, showing excellent validity in predicting in-hospital mortality (AUCs > 0.900) and at least very good validity for short-term outcomes (AUCs > 0.820, Supplementary Table).

For the long-term mortality and outcome, in most occasions the short scales showed very good validity (AUCs = 0.814–0.893 for mortality, 0.808–0.901 for mRS 3–6, and 0.794–0.928 for GOSE 1–4, Supplementary Table). The GCS validity was higher, and it was found to be excellent (> 0.900) without any exception (Supplementary Table).

To summarize, there was a clear trend towards a higher GCS validity for all outcomes and raters since all AUCs were greater than those for the three short scales. No remarkable difference between the prognostic validity of the scales for trauma patients compared to the whole sample was seen.

## Discussion

In the present study, the validity of three simplified consciousness scales in identifying coma and predicting short- and long-term outcome in neurosurgical patients was assessed. The results indicate that the short scales showed in general good prognostic validity for all outcomes of interest, yet lower than the GCS in all cases. This general trend remained unchanged in the sub-group analysis for head trauma patients.

The GCS, the globally applied clinical tool for assessing consciousness, has received criticism since the first years of its use in clinical practice. However, this critique has not been consistent, especially regarding its complexity. The notion that it is unnecessarily complicated has led to the development of simpler assessing systems [8, 10, 13, 23, 29]. On the contrary, it has also been postulated that it omits crucial information [26]. Thus, a comparison between the prognostic validity of the GCS and simpler, easier-applied scales is of particular interest. The fact that the prognostic validity of the simpler scales was overall lower than the GCS was to some extent anticipated since these scales contain similar clinical data, but they provide fewer details than the GCS. It also indicates that the comparison between simpler coma scales and the GCS in a statistically sound way is of some significance.

In an effort to assess their importance in clinical practice, previous works estimated the validity of simpler scales in predicting neurosurgical intervention or intubation [8, 13, 23, 29, 30]. Nevertheless, any attempt to link the need for medical and surgical interventions with the level of consciousness lacks scientific justification, since severe disturbance of consciousness is frequently not the sole indication for intubation [39]. Further, the decision to proceed to a neurosurgical intervention is multifactorial, and a severe consciousness disturbance might render surgery pointless [40, 41]. On the contrary, discriminating comatose patients is fundamental. Thus, the scales’ validity in identifying coma is a preferable way to assess their clinical importance, because these clinical tools are specifically designed to assess consciousness.

As already mentioned, according to the current results, the prognostic validity of the simplified scales was overall acceptable, but lower than that of the GCS and frequently not even comparable. Thus, there are many reasons to question a potential replacement of the GCS in clinical practice [8, 13]. Contrary to what has been supported in the literature [29], the ability of a straightforward assessment has been a well-established advantage of the GCS [3, 4], a scale on the application of which healthcare professionals already have long-standing experience. Moreover, it has been shown that proper training is sufficient to improve the raters’ agreement, even for more complicated scales [42].

It is worth mentioning that the inter-rater reliability of the simplified scales has not been well-studied. Available reports are limited, with some not even assessing the more clinically sound weighted kappa index [9]. It is, also, surprising that in previous reports, where the validity of the simpler scales was assessed, no comparison with the GCS in a statistically sound way was performed, with the limit of significant difference arbitrarily defined [8, 12, 13, 30]. When it comes to AVPU it becomes even more confusing, since the scale’s categories are not even clearly defined and quantified, with important differences between studies [10, 13]. Interestingly, McNarry et al., instead of training their staff in the use of the scale, encouraged them to use “their own judgement” [11], possibly referring to an intuitive assessment. Another simplified assessment model, the ACDU scale, was also excluded from the present study for a similar reason [9, 11].

Therefore, it can be stated that any potential superiority of less detailed scales in clinical practice is far from proven and better training on the implementation of the GCS might be more valuable. Considering the possibility of elimination of potentially crucial information on the patient’s condition and assessing its impact on consciousness monitoring, there is a need to conduct well-designed clinimetric studies on those scales. Notably, this has also been a point of criticism for the GCS, and a reason that led to the development of even more complex and comprehensive scales, such as the FOUR Score [26].

The study has some limitations. It is a one-center analysis, restricted to neurosurgical patients. Although the inclusion criteria were clearly defined (pathology of neurosurgical interest and need for consciousness assessment and monitoring) and head trauma was the most frequent underlying pathology, the sample was to some extent inhomogeneous. It has to be stated, however, that research on consciousness assessment scales frequently includes a variety of underlying diagnoses causing acute brain insult [9, 11, 21, 26]. Α sub-group analysis for trauma cases that provided similar results was performed, but the sample size was not sufficient to allow reliable comparisons for the prognostic value of the scales. The assessments were performed with the GCS and the values of the other scales were calculated based on those recordings. Even though this is an accepted methodology [8, 12, 13, 23], statistical parameters such as inter-rater reliability could not be estimated. The aim of the study was to compare the validity of each scale with the GCS, and not with each other. However, given the fact that the current results do not support the substitution of the GCS with any of the tested scales, such a comparison wasn’t considered meaningful. AUCs were estimated for the prediction of mortality and functional outcome, which is a methodology repeatedly used in similar clinimetric studies [20, 21, 28, 30], but it must be stressed that the level of consciousness cannot be used as a sole predictor for the outcome. Thus, confounding factors, such as comorbidities or neurologic sequelae, might have affected the patients’ outcome, although in the present study to a minimal extent, since the neurological deficits in all included cases were caused by the main brain insult.

Nevertheless, this is a prospective study with a rigorous design, which provides an in-depth analysis of the prognostic value of three simplified clinical tools for the evaluation of the level of consciousness. The follow-up period was long, no patient was lost, and four healthcare professionals with different experience and background performed the assessments. Further, the role of a number of scales in diagnosing coma was thoroughly assessed and an in-depth comparison with the GCS was also done.

## Conclusions

The validity of the three simplified consciousness assessment scales that were studied was high but inferior to that of the GCS. Thus, their actual clinical utility remains questionable. A number of additional concerns regarding their usefulness in clinical practice exist, in particular related to patient monitoring. Therefore, the GCS still remains the most important and reliable tool for the assessment of disorders of consciousness.

## Data Availability

The datasets used and analyzed in this study are available on reasonable request.

## References

[1] Teasdale G, Jennett B (1974). Assessment of coma and impaired consciousness. A practical scale. Lancet.

[2] Teasdale G, Jennett B (1976). Assessment and prognosis of coma after head injury. Acta Neurochir (Wien).

[3] Kornbluth J, Bhardwaj A (2011). Evaluation of coma: a critical appraisal of popular scoring systems. Neurocrit Care.

[4] Matis G, Birbilis T (2008). The Glasgow Coma Scale—a brief review. Past, present, future. Acta Neurol Belg.

[5] Laureys S, Bodart O, Gosseries O (2014). The Glasgow Coma Scale: time for critical reappraisal?. Lancet Neurol.

[6] Moore SA, Wijdicks EF (2013). The acutely comatose patient: clinical approach and diagnosis. Semin Neurol.

[7] Laureys S, Piret S, Ledoux D (2005). Quantifying consciousness. Lancet Neurol.

[8] Gill M, Steele R, Windemuth R, Green SM (2006). A comparison of five simplified scales to the out-of-hospital Glasgow Coma Scale for the prediction of traumatic brain injury outcomes. Acad Emerg Med.

[9] Gill M, Martens K, Lynch EL, Salih A, Green SM (2007). Interrater reliability of 3 simplified neurologic scales applied to adults presenting to the emergency department with altered levels of consciousness. Ann Emerg Med.

[10] Kelly CA, Upex A, Bateman DN (2004). Comparison of consciousness level assessment in the poisoned patient using the alert/verbal/painful/unresponsive scale and the glasgow coma scale. Ann Emerg Med.

[11] McNarry AF, Goldhill DR (2004). Simple bedside assessment of level of consciousness: comparison of two simple assessment scales with the Glasgow Coma scale. Anaesthesia.

[12] Eftekhar B, Zarei MR, Ghodsi M, MoezArdalan K, Zargar M, Ketabchi E (2005). Comparing logistic models based on modified GCS motor component with other prognostic tools in prediction of mortality: results of study in 7226 trauma patients. Injury.

[13] Hopkins E, Green SM, Kiemeney M, Haukoos JS (2018). A two-center validation of "Patient Does Not Follow Commands" and three other simplified measures to replace the glasgow coma scale for field trauma triage. Ann Emerg Med.

[14] Green SM (2011). Cheerio, laddie! bidding farewell to the Glasgow Coma Scale. Ann Emerg Med.

[15] Von Elm E, Altman DG, Egger M, Pocock SJ, Gøtzsche PC, Vandenbroucke JP (2007). The Strengthening the Reporting of Observational Studies in Epidemiology (STROBE) statement: guidelines for reporting observational studies. Ann Intern Med.

[16] Rankin J (1957). Cerebral vascular accidents in patients over the age of 60: III. Diagnosis and treatment. Scott Med J.

[17] Van Swieten J, Koudstaal P, Visser M, Schouten H, Van Gijn J (1988). Interobserver agreement for the assessment of handicap in stroke patients. Stroke.

[18] Jennett B, Bond M (1975). Assessment of outcome after severe brain damage: a practical scale. The Lancet.

[19] Jennett B, Snoek J, Bond M, Brooks N (1981). Disability after severe head injury: observations on the use of the Glasgow Outcome Scale. J Neurol Neurosurg Psychiatry.

[20] Sadaka F, Patel D, Lakshmanan R (2012). The FOUR score predicts outcome in patients after traumatic brain injury. Neurocrit Care.

[21] Chen B, Grothe C, Schaller K (2013). Validation of a new neurological score (FOUR Score) in the assessment of neurosurgical patients with severely impaired consciousness. Acta Neurochir (Wien).

[22] Frowein R (1976). Classification of coma. Acta Neurochir.

[23] Haukoos JS, Gill MR, Rabon RE, Gravitz CS, Green SM (2007). Validation of the Simplified Motor Score for the prediction of brain injury outcomes after trauma. Ann Emerg Med.

[24] Brunker C, Harris R (2015). How accurate is the AVPU scale in detecting neurological impairment when used by general ward nurses? An evaluation study using simulation and a questionnaire. Intensive Crit Care Nurs.

[25] Anestis DM, Foroglou NG, Varoutis PC, Monioudis PM, Tsonidis CA, Tsitsopoulos PP (2023). Comparison of the prognostic value of coma scales among health-care professionals a prospective observational study. Acta Neurol Belg..

[26] Wijdicks EF, Bamlet WR, Maramattom BV, Manno EM, McClelland RL (2005). Validation of a new coma scale: the FOUR score. Ann Neurol.

[27] Wolf CA, Wijdicks EF, Bamlet WR, McClelland RL (2007). Further validation of the FOUR score coma scale by intensive care nurses. Mayo Clin Proc.

[28] Okasha AS, Fayed AM, Saleh AS (2014). The FOUR score predicts mortality, endotracheal intubation and ICU length of stay after traumatic brain injury. Neurocrit Care.

[29] Singh B, Murad MH, Prokop LJ, Erwin PJ, Wang Z, Mommer SK (2013). Meta-analysis of Glasgow coma scale and simplified motor score in predicting traumatic brain injury outcomes. Brain Inj.

[30] Chou R, Totten AM, Carney N, Dandy S, Fu R, Grusing S (2017). Predictive utility of the total Glasgow coma scale versus the motor component of the Glasgow coma scale for identification of patients with serious traumatic injuries. Ann Emerg Med.

[31] de Long ER, de Long DM, Clarke-Pearson DL (1988). Comparing the areas under two or more correlated receiver operating characteristic curves: a nonparametric approach. Biometrics.

[32] Mandrekar JN (2010). Receiver operating characteristic curve in diagnostic test assessment. J Thorac Oncol.

[33] Mansour OY, Megahed MM, Abd Elghany EHS (2015). Acute ischemic stroke prognostication, comparison between Glasgow Coma Score, NIHS Scale and Full Outline of UnResponsiveness Score in intensive care unit. Alex J Med.

[34] Wijdicks EF, Kramer AA, Rohs T, Hanna S, Sadaka F, O'Brien J (2015). Comparison of the Full Outline of UnResponsiveness score and the Glasgow Coma Scale in predicting mortality in critically ill patients. Crit Care Med.

[35] Said T, Chaari A, Hakim KA, Hamama D, Casey WF (2016). Usefulness of full outline of unresponsiveness score to predict extubation failure in intubated critically-ill patients: a pilot study. Int J Crit Illn Inj Sci.

[36] Bland JM, Altman DG (1995). Multiple significance tests: the Bonferroni method. BMJ.

[37] Saito M, Seo Y, Yano Y, Momose K, Hirano H, Yoshida M (2013). Reduction in non-protein respiratory quotient is related to overall survival after hepatocellular carcinoma treatment. PLoS ONE.

[38] Anestis DM, Monioudis PM, Foroglou NG, Tsonidis CA, Tsitsopoulos PP (2022). Clinimetric study and review of the reaction level scale. Acta Neurolog Scand.

[39] Fulton JA, Greller HA, Hoffman RS (2005). GCS and AVPU: the alphabet soup doesn't spell “COMA” in toxicology. Ann Emerg Med.

[40] Anderson E, Kryzanski J (2020). Prognosis and futility in neurosurgical emergencies: a review. Clin Neurol Neurosurg.

[41] Honeybul S, Gillett GR, Ho K (2013). Futility in neurosurgery: a patient-centered approach. Neurosurgery.

[42] Anestis DM, Tsitsopoulos PP, Foroglou NG, Tsatali MS, Marinos K, Theologou M (2021). Cross-cultural adaptation and validation of the greek version of the “Full Outline of Unresponsiveness Score”: a prospective observational clinimetric study in neurosurgical patients. Neurocrit Care.

